# The Essential Role of Mbd5 in the Regulation of Somatic Growth and Glucose Homeostasis in Mice

**DOI:** 10.1371/journal.pone.0047358

**Published:** 2012-10-15

**Authors:** Yarui Du, Bo Liu, Fan Guo, Guifang Xu, Yuqiang Ding, Yong Liu, Xin Sun, Guoliang Xu

**Affiliations:** 1 The State Key Laboratory of Molecular Biology, Institute of Biochemistry and Cell Biology, Shanghai Institutes for Biological Sciences, Chinese Academy of Sciences, Shanghai, People’s Republic of China; 2 Tongji University School of Medicine, Shanghai, People’s Republic of China; 3 Institute of Nutrition Sciences, Shanghai Institutes for Biological Sciences, Chinese Academy of Sciences, Shanghai, People’s Republic of China; 4 Laboratory of Genetics, University of Wisconsin-Madison, Madison, Wisconsin, United States of America; Hosptial Infantil Universitario Niño Jesús, CIBEROBN, Spain

## Abstract

Methyl-CpG binding domain protein 5 (MBD5) belongs to the MBD family proteins, which play central roles in transcriptional regulation and development. The significance of MBD5 function is highlighted by recent studies implicating it as a candidate gene involved in human 2q23.1 microdeletion syndrome. To investigate the physiological role of Mbd5, we generated knockout mice. The Mbd5-deficient mice showed growth retardation, wasting and pre-weaning lethality. The observed growth retardation was associated with the impairment of GH/IGF-1 axis in Mbd5-null pups. Conditional knockout of Mbd5 in the brain resulted in the similar phenotypes as whole body deletion, indicating that Mbd5 functions in the nervous system to regulate postnatal growth. Moreover, the mutant mice also displayed enhanced glucose tolerance and elevated insulin sensitivity as a result of increased insulin signaling, ultimately resulting in disturbed glucose homeostasis and hypoglycemia. These results indicate Mbd5 as an essential factor for mouse postnatal growth and maintenance of glucose homeostasis.

## Introduction

In vertebrates, cytosine methylation in DNA is one of the major epigenetic modifications, which regulates many cellular events, including developmental gene expression, X chromosome inactivation, genome defense, and genomic imprinting [1]. DNA methylation exerts regulatory functions by recruiting specific binding proteins that contain a highly conserved methyl-CpG binding domain (MBD) [2]. Five mammalian MBD family proteins, MeCP2, MBD1, MBD2, MBD3 and MBD4, have been well characterized. These proteins, except for MBD3, bind selectively to methylated DNA [3], [4] and play roles in transcriptional repression and chromatin remodeling [5], [6], [7]. The developmental significance of MBD proteins in interpreting DNA methylation patterns and mediating transcriptional repression has been demonstrated mainly in human congenital disorders and knockout mouse models [8].

Based on homology searches using the conserved MBD domain, an additional member, termed MBD5, was identified [9], [10]. Little is known about the function of MBD5. In addition to the MBD domain, MBD5 also harbors a PWWP domain. This domain is also found in DNA methyltransferase DNMT3B and the mutation of DNMT3B causes ICF immunodeficiency syndrome [11]. In cultured cells, the MBD5 protein associates with heterochromatin, although it cannot directly bind to methylated DNA [12]. Several lines of evidence have suggested that MBD*5* is a single causal locus of human mental disorders. First, microdeletions of the *MBD5* gene were detected in 65 patients with mental retardation [13], [14], [15], [16], [17], [18]. Second, four low-frequency missense variants in the coding sequence of *MBD5* were found in mentally retarded patients but not in healthy controls [14]. Finally, the *MBD5* gene is located on chromosome 2q23.1, a region in which aberrations are associated with epilepsy [19]. Based on the presence of the MBD and PWWP domains in the encoded protein and the association of *MBD5* mutation with human mental retardation, we hypothesized that MBD5 plays a unique role during development.

In this study, we aimed to define the *in vivo* function of *Mbd5* through the generation and characterization of the knockout mice. The whole body Mbd5 knockout mice displayed severe growth retardation and highly penetrant pre-weaning lethality. The impairment of GH/IGF-1 axis potentially contributes to the observed growth retardation in the mutant mice. Furthermore, the similar phenotypes observed in the brain-specific knockout mice reveal that Mbd5 functions in the nervous system to regulate postnatal growth. The knockout mice also showed persistent hypoglycemia, hypoinsulinemia, enhanced glucose tolerance and elevated insulin sensitivity. Overall, Mbd5 plays an essential role in the control of postnatal growth and glucose homeostasis.

## Results

### Generation of Mbd5-deficient Mice

**Figure 1:**
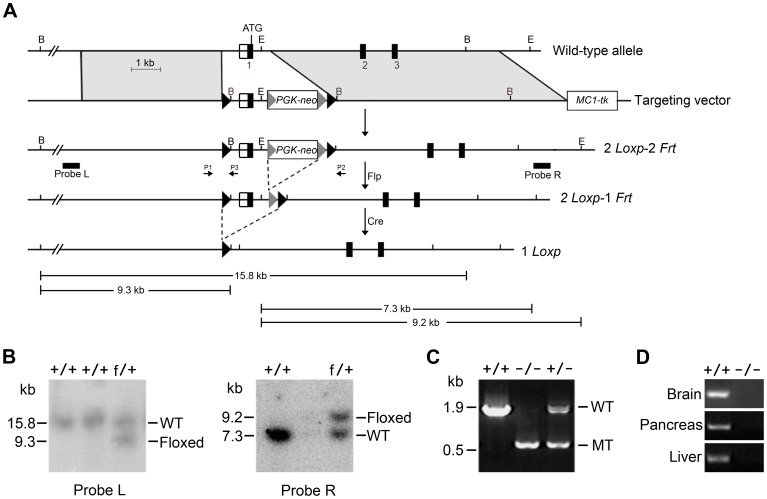
Targeted disruption of *Mbd5* in mice. (**A**) The strategy for the generation of a targeted *Mbd5* allele. Numbered black boxes represent the coding exons and open boxes represent the 5′ untranslated region (UTR). The *Lox*P and *Frt* sites are shown as black and gray triangles, respectively. The floxed region contains exon 1, which encodes the majority of the MBD domain. The probes used in the Southern blot analysis to correctly identify targeted ES cells are indicated by the horizontal bars. The restriction sites used to digest ES genomic DNA were *Bam*HI (B) and *Eco*RV (E). The PCR primers used for genotyping are indicated with arrows P1, P2 and P3. (**B**) Identification of targeted ES clones by Southern blot analysis using two different probes. (**C**) Verification of the mutant allele in homozygous and heterozygous mice by genomic PCR with primers P1 and P2. (**D**) Loss of *Mbd5* mRNA in knockout mice. RNA samples from the brain, pancreas and liver were examined by RT-PCR. The forward primer used was located in the targeted genomic region. A targeted allele following Cre-deletion generated no PCR product, and the floxed allele generated a 305-bp product.

To investigate the physiological function of *Mbd5*, we generated knockout mice by gene targeting. The targeted genomic region, which was flanked by *lox*P sites, included the first exon encompassing the 5′UTR and the majority of the MBD domain coding region (Figure 1A). Homologous recombination at the desired locus in the selected ES cell lines was confirmed by Southern blot analysis with two different external probes, respectively (Figure 1B). Chimeras, which were derived from blastocysts with the injection of positive ES cells, were crossed with C57BL/6 mice. The F1 mice carrying a floxed allele were crossed with *EIIa*-Cre mice to delete the floxed region in the *Mbd5* gene. Heterozygotes were intercrossed to generate homozygotes. The genotypes of all mice were confirmed by PCR (Figure 1C) and the absence of full-length *Mbd5* mRNA in homozygous mutants was confirmed in various tissues by RT-PCR analysis (Figure 1D).

### Mbd5-deficient Mice Show Early Postnatal Lethality with Severe Growth Retardation

**Figure 2:**
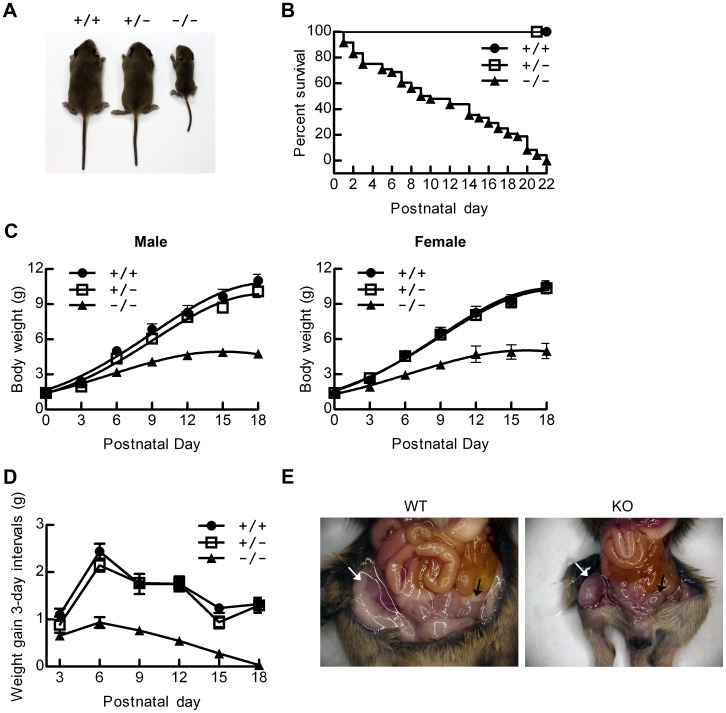
Postnatal growth retardation and pre-weaning lethality of *Mbd5^−/−^* mice. (**A**) Gross morphology of typical wild-type (+/+), heterozygous (+/−) and homozygous (−/−) mice at postnatal day 14 (P14). The Mbd5-knockout mice had significantly smaller body size than their littermates, and the reduction in body weight was accompanied by a reduction in body length. (**B**) Survival curve of *Mbd5^−/−^* pups (n = 48) and their littermate controls. (**C**) Growth curve of *Mbd5^−/−^* pups (n = 4–5 for each gender) and their littermate controls (For WT, n = 4–5 for each gender, for heterozygotes, n = 9–10 for each gender). The offspring generated from heterozygous intercrosses of *Mbd5^+/−^* mice were weighed at 3-day intervals as indicated. (**D**) Growth gain curve of *Mbd5^−/−^* mice (n = 9) and their littermate controls (8 wild-type and 19 heterozygotes). The growth rates shown were calculated by subtracting the value of cumulative weight at a particular day from that of three days before, i.e., each point represents the weight gain of the three preceding days. (**E**) Reduction of subcutaneous fat (white arrows) and perigonadal fat (black arrows) in Mbd5-knockout mouse at P14.

The interbreeding of heterozygous mice yielded F2 offspring of *Mbd5^+/+^*, *Mbd5^+/−^* and *Mbd5^−/−^* at an approximately expected Mendelian ratio (68∶131∶49), indicating that *Mbd5* deficiency does not lead to embryonic lethality. Although the heterozygotes appeared grossly normal and were fertile (Figure 2A–C, S1A, Table S1), all Mbd5*-*deficient mice suffered severe growth retardation (Figure 2A) and died prior to weaning between postnatal day (P) 1 and 22 (Figure 2B). A comparable amount of milk was present in the stomachs of dead knockout pups and their sacrificed normal littermates (Figure S1B), indicating that the lethality didn’t result from a feeding defect. As shown in Figure 2A, the surviving null mice were wasting and had decreased axial growth. The body weight of homozygotes was comparable to either heterozygotes or wild-type mice at birth, but became noticeably lower within 3 days after birth and was greatly reduced one week after birth. In contrast to the controls, the weight gain of the mutant mice plateaued starting at two weeks after birth (Figure 2C). Similar phenotypes were observed in both genders (Figure 2C), and therefore we pooled the groups in the following experiments except where otherwise noted. For 3-day weight gain of normal mice, there were two growth rate peaks before weaning. The first peak was at approximately P6 and the second growth spurt commenced at approximately two weeks; the second time point coincides with the initiation of growth hormone action [20]. However, the null mice lacked the second growth peak and their first peak was much lower than that of the control mice (Figure 2D). We also observed that the weights of major mutant organs determined at 1 week of age were reduced proportional to body weights in comparison with wild-type controls, with the exception of brain and liver (Table 1). The liver was disproportionately reduced in size, whereas the brain/body weight ratio was relatively increased by about 80% in mutants. Most likely, this difference is due to the fact that brain growth is practically completed by the time GH action commences in mice [20]. The higher ratio of brain in Mbd5 KO mice is due to their growth retardation (smaller denominator). Along with the growth retardation, the Mbd5-knockout mice also displayed a reduction in perigonadal and subcutaneous fat compared with the control mice (Figure 2E). Overall, these results demonstrate that Mbd5 is essential for postnatal survival and growth.

**Table 1:** Comparison of organ weights between wild-type and *Mbd5^−/−^* mice.

	Weight (mg)		% of body weight (% of control)		
Organ	WT	KO	WT	KO	P value
Brain	308.1±14.06	225.9±16.77	5.28±0.19 (100±3.6)	9.67±0.53 (182.9±5.7)	<0.0001
Thymus	37.1±2.45	11.4±2.32	0.54±0.02 (100±3.3)	0.46±0.04 (86.8±8.3)	0.19
Heart	33.0±2.00	13.8±2.37	0.56±0.01 (100±2.5)	0.57±0.03 (100.9±5.9)	0.9
Lung	91.6±5.00	38.4±4.41	1.59±0.07 (100±4.5)	1.79±0.06 (112.4±2.5)	0.08
Liver	174.9±10.28	60.8±7.71	3.01±0.13 (100±4.5)	2.55±0.03 (83.2±5.0)	0.047
Pancreas	19.9±1.70	7.6±1.84	0.34±0.04 (100±10.3)	0.31±0.04 (93.8±6.3)	0.63
Spleen	38.0±5.23	11.5±3.79	0.62±0.09 (100±14.5)	0.42±0.10 (67.4±8.6)	0.12
Kidney	69.8±6.19	31.4±3.12	1.19±0.06 (100±5.4)	1.33±0.04 (112.3±3.3)	0.10
Body (g)	5.9±0.35	2.4±0.30			

Data are means ± SEM for four matched male mice at P7. Relative (% of control) values ± SEM were compared to evaluate statistically significant differences.

### Mbd5 Deficiency Leads to Reduced Somatotropic Signaling

**Figure 3:**
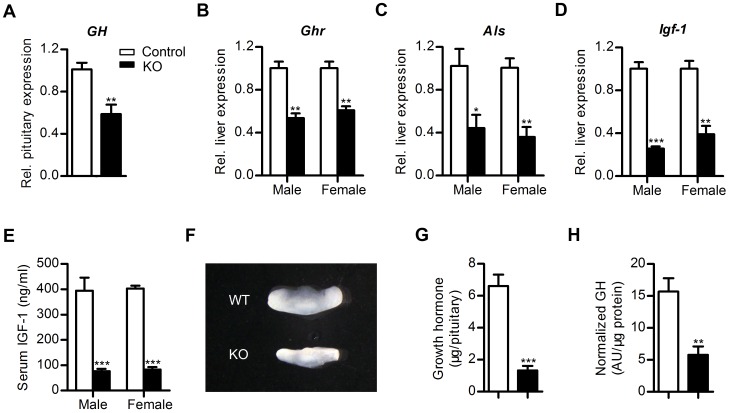
Reduced somatotropic signaling in Mbd5-knockout mice. (**A**) Pituitary expression of *GH* in control and knockout mice. *Gapdh* was used for normalization. Gene expression levels were determined by real-time PCR in P14 mice. At least 5 pairs of matched female mice were used for the comparison. (**B–D**) RNA levels of *Ghr*, *Als* and *Igf-1* in the liver of control and knockout mice at P14. *β-actin* was used for normalization. n = 3 per group. (**E**) Serum IGF-1 concentrations in P14 mice. For each group, n = 5–6. (**F**) Pituitary from KO and wild-type littermates at P14. (**G, H**) Pituitary GH stores (**G**) and GH content normalized to pituitary protein level (**H**) in 2-week-old Mbd5 KO mice and their littermate controls. n = 4 per group. *, P<0.05, **, P<0.01; ***, P<0.001.

To further dissect the mechanism underlying reduced growth, we directly examined somatotropic signaling, which is critical for postnatal growth and for directing elongation of the long bones in animals [21]. As the Mbd5*-*deficient mice lacked the second growth peak, a peak induced by the action of GH commencing at 2 weeks after birth in normal mice, we used 2-week-old mice for further somatotropic analysis. The mRNA expression level of *GH* in the pituitary was decreased by about 42% in the mutant mice (Figure 3A). Despite considerable variation of serum GH concentrations presumably due to the pulsatile secretion pattern of GH [22], the knockout mice still showed a trend of reduction in circulating GH concentration (Figure S2A). The growth hormone releasing hormone receptor (Ghrhr) and the ghrelin receptor (Ghsr) act as the stimulatory receptors of GH release and the somatostatin receptors (Sstr2, Sstr5) act as the inhibitory receptors of GH release. The expression levels of these genes in the mutant pituitary were unchanged (Figure S2B). GH acts on the GH receptors (Ghr) in the liver, inducing transcription and secretion of insulin-like growth factor 1 (IGF-1) and IGF binding protein acid-labile subunit (ALS) [23]. The levels of IGF-1 and ALS are positively correlated with growth [24], [25]. Along with decreased GH level, the transcription levels of *Ghr*, *Als* and *Igf-1*, which are downstream of GH action in the liver, were reduced (Figure 3B–D). Consistently, the serum IGF-1 protein level was reduced to 20% of the wild-type level (Figure 3E). More importantly, the reduction of serum IGF-1 level was already obvious in newborn mutant mice (Figure S3B), before size reduction is apparent. The expression level of *Igf-1* was only reduced in liver (Figure 3D, Figure S3A), which contributed to reduced circulating IGF-1 levels in the mutant mice (Figure 3E).

Since GH is synthesized, stored, and released by the pituitary, we next examined this gland in the knockout mice. Pituitaries from Mbd5 KO mice were smaller than those of wild-type littermates (Figure 3F), but displayed no obvious structural abnormalities by H&E staining (Figure S2C). Furthermore, each pituitary gland contained significantly less stored GH in KO mice as compared with controls (Figure 3G), even when the smaller pituitary size of knockout mice was taken into account (Figure 3H). To determine whether the defect in GH reflected a more general deficit in pituitary hormone production, we measured mRNA levels of two additional pituitary hormones–the thyroid-stimulating hormone (*TSHβ*) and the adrenocorticotropic hormone (*ACTH*). The expression levels of both genes were unchanged (Figure S2D). Thus, the pituitary defect of KO mice appears to be restricted to GH. Taken together, Mbd5-deficient mice show decreased pituitary GH stores, and subsequently reduced IGF-1 serum levels, and ultimately impaired somatic growth, revealing a critical role for Mbd5 in control of somatic growth.

### Neural-specific Mbd5 Knockout Mice also Show Growth Retardation and Preweaning Lethlatity

**Figure 4:**
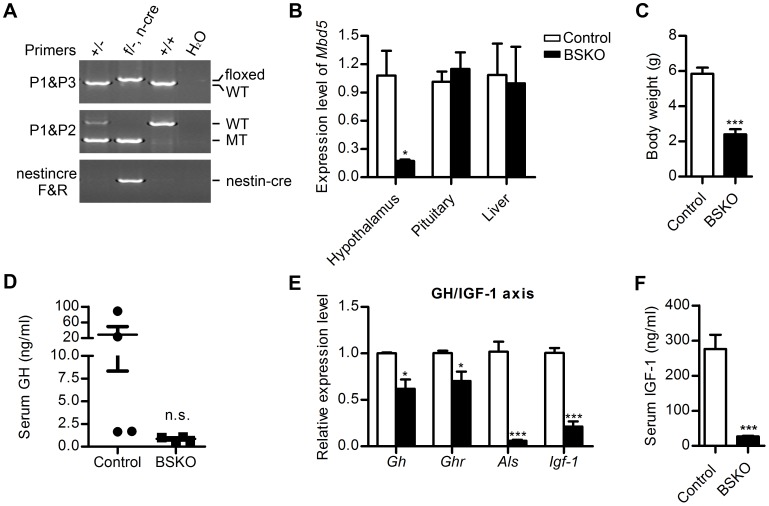
Specific deletion of Mbd5 in brain results in similar phenotypes as the whole body deletion. (**A**) Genotype confirmation of Mbd5 brain-specific knockout mice by PCR with primers indicated. (**B**) *Mbd5* mRNA level in different tissues of Mbd5 BSKO mice normalized to the control levels. *Gapdh* was used for normalization. At least 3 pairs of matched mice were used for comparison. (**C**) Body weight of Mbd5 BSKO mice and their littermates controls at P7. n = 4 per genotype. (**D**) Serum GH concentrations in 2-week-old BSKO and littermate controls. n = 4 per genotype. (**E**) Expression levels of GH/IGF-1 axis associated genes in Mbd5 BSKO mice and littermate controls at P14 as measured by real-time PCR. *β-actin* was used for normalization. n = 3 per group. (**F**) Serum IGF-1 concentrations in P14 mice. At least 6 pairs of matched mice were used for comparison. *, P<0.05; ***P<0.001.

Because development and function of the pituitary are under the control of the hypothalamus [26], we hypothesize that the pituitary defects observed in KO mice are due to alterations in hypothalamic function in the absence of Mbd5. Therefore, we generated brain-specific Mbd5 knockout mice (BSKO, [*Mbd5^f/−^*, Nestin-Cre]) by crossing *Mbd5^f/f^* mice with the Nestin-Cre transgenic mice [27] (Figure 4A). Real-time PCR analysis showed that Nestin-Cre mediated deletion of Mbd5 was confined to the neuronal lineage (Figure 4B). Similar to whole body deletion, the BSKO mice also showed growth retardation (Figure 4C) and pre-weaning lethality (data not shown). In addition, the BSKO mice displayed defects in somatotropic axis with lower serum GH and IGF-1 levels (Figure 4D–F). These observations support a central role of neural Mbd5 in somatic growth regulation and postnatal survival. Overall, our findings show that loss of Mbd5 compromises the somatotropic axis, which may be caused by impaired hypothalamus function.

### Mbd5-deficient Mice Showed Disturbance of Glucose Homeostasis Due to Elevated Insulin Sensitivity

**Figure 5:**
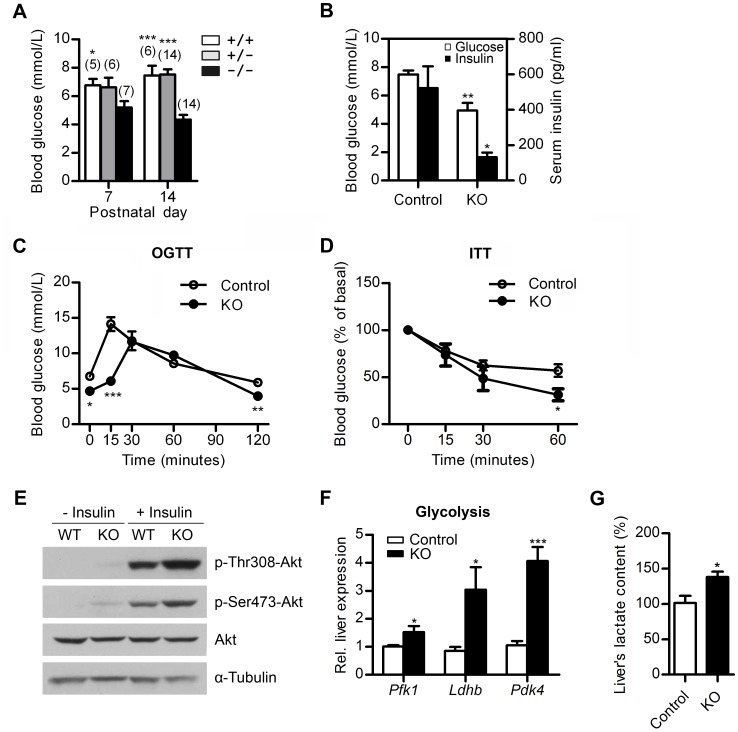
Disturbed glucose homeostasis with elevated insulin sensitivity in *Mbd5^−/−^* mice. (**A**) Blood glucose levels in WT, *Mbd5^+/−^* and *Mbd5^−/−^* mice in the fed state at the indicated ages. The numbers in brackets indicate the number of animals studied. (**B**) Blood glucose and serum insulin levels of 2-week-old mice in the fed state. For each group, n = 5. (**C**) The OGTT of control and Mbd5-knockout male mice at P14. For each group, n = 5. (**D**) The ITT of control and Mbd5-knockout male mice at P14. For each group, n = 5. *, P<0.05; **, P<0.01; ***, P<0.001. (**E**) Akt activation in the liver of control and Mbd5-knockout mice at P14. The protein levels were analyzed by western blotting with the antibodies indicated. α-tubulin was used as a loading control. (**F**) Alteration in the mRNA levels of glycolytic genes in the livers of Mbd5-knockout mice at P14. The expression of each gene was normalized to *β-actin*. At least 5 pairs of matched mutant and wild-type mice were used for comparison. (**G**) Lactate levels in the livers of Mbd5-knockout mice and controls at P14. n = 3 per genotype *, P<0.05; **, P<0.01; ***, P<0.001.

Somatic growth and insulin sensitivity are interlinked because a reduction in somatic growth by genetic methods improves glucose homeostasis and insulin sensitivity in mice, rats and humans [28], [29], [30]. We next addressed whether glucose homeostasis and insulin sensitivity were altered. Compared with the wild-type and heterozygous littermates, the blood glucose levels of homozygous mutant pups were 20% and 40% lower at one- and two-weeks of age, respectively (Figure 5A). Concomitantly, the serum insulin levels in the knockout mice in the ad libtum-fed state were reduced by approximately 60% compared with control littermates (Figure 5B). No alteration in pancreatic morphology was observed (Figure S5A), and the distribution of glucagon- and insulin-expressing cells (Figure S5B) as well as the pattern of glucose sensing protein transporter GLUT2 (Figure S5C) in pancreatic islets were indistinguishable between the mutant and wild-type mice. Thus, we speculated that the lower blood glucose might have triggered a reduction in insulin secretion as an adaptive response. We also assessed the performance of control and knockout mice in oral glucose tolerance tests (OGTT) and insulin tolerance tests (ITT). After glucose was administered orally, the mutant mice cleared glucose faster than the control animals at 60 min and thereafter, although the mutant mice displayed a delay in glucose absorption (Figure 5C). Since the fasted glucose level in the mutant mice was lower than controls’ (Figure 5C, S4A), we set the basal glucose at time 0 at the same level (100%), and found the OGTT between KO and control mice was truly different (Figure S4B), further indicating enhanced glucose tolerance in the KO mice. The mutant mice also exhibited a significantly improved performance in the ITT (Figure 5D). To further identify the tissue mainly responsible for altered glucose homeostasis, we then determined the liver insulin sensitivity because the hepatic glucose production is under the direct hepatic control of insulin action. Western blot analyses showed that pAkt levels of both pAkt-S473 and -T308 were increased with or without insulin treatment in the Mbd5-deficient livers compared with controls (Figure 5E). These data indicate that Mbd5-knockout mice are more sensitive to insulin. Consistent with insulin hypersensitivity, the knockout mice displayed enhanced liver glycolysis as evidenced by the up-regulation of metabolic genes *Pfk1*(rate-limiting glycolytic enzyme phosphofructokinase 1), *Ldhb* (lactate dehydrogenase B) and *Pdk4* (pyruvate dehydrogenase kinase 4) as well as a higher level of liver lactate (Figure 5F, G). Overall, the elevated insulin sensitivity in dwarf Mbd5-deficient mice might contribute to the alteration of glucose homeostasis, resulting in hypoglycemia.

## Discussion

In this study, we identified the *in vivo* function of a novel MBD family protein, MBD5, in mice. Disruption of Mbd5 in mice resulted in postnatal growth retardation that manifested as reduced body weight, body length and impaired GH/IGF-1 signaling. Moreover, brain- specific deletion of Mbd5 resulted in the similar phenotypes as whole body deletion, indicating that Mbd5 functions in the brain to regulate postnatal growth. Interestingly, the mutant mice also showed altered glucose homeostasis due to elevated insulin sensitivity. Our work reveals a potential critical role of Mbd5 in postnatal growth and glucose homeostasis.

Several MBD family proteins play important roles in the development and function of the nervous systems [8]. Mutations in methyl-CpG binding protein 2 (MeCP2) are linked to a human mental retardation disorder, the Rett syndrome [31]. *Mbd1^−/−^* mice are healthy and fertile but exhibit decreased neurogenesis, impaired spatial learning, and increased genome instability in adult neural stem cells [32]. *Mbd2^−/−^* mice appear normal but exhibit a defect in maternal nursing behavior with an unknown cause [33]. A follow-up study by an international consortium recently revealed that *MBD5* was a single causal locus in 65 human patients with the 2q23.1 microdeletion syndrome. The syndromic features of 2q23.1 microdeletion syndrome involve developmental delays, growth retardation, microcephaly, behavioral problems, seizures and intellectual disabilities [18]. Our Mbd5-deficient mice exhibit phenotypes that resemble some of syndromes of human patients with 2q23.1 microdeletion, including growth retardation (Figure 2A, C) and absolute smaller brain size (Figure S1C, Table 1). But we didn’t see apparent behavioral problems except that the knockout mouse always lowered its head when moving forward (Movie S1, S2) likely due to its higher brain ratio (Table 1). Although there is a haploinsufficient phenotype in the human population, the Mbd5 mouse heterozygotes appeared grossly normal and were fertile (Figure 2A, S1A, Table S1). The functional pathway of Mbd5 between mouse and human could be different, or the 2q23.1 microdeletion in human involves the combination of *MBD5* and other genes.

Postnatal growth retardation that is associated with impaired GH/IGF-1 signaling is a prominent feature of Mbd5 mutant mice (Figure 3A–E). Because brain homeostatic centers control somatic growth and IGF-1 levels, and *Mbd5* is highly expressed in the central nervous system [12], we hypothesize that the loss of neural *Mbd5* might contribute to the growth defects of the mutant mice. In accordance with this hypothesis, specific deletion of Mbd5 in brain results in similar phenotypes as whole body deletion (Figure 4), indicating the phenotype is caused by Mbd5 deficiency in the nervous system rather than in peripheral tissues. The mechanism of Mbd5 function in brain to regulate postnatal growth warrants further investigation.

As has been shown in multiple studies of dwarfism, mice with reduced levels of GH and IGF1 are insulin sensitive [28], [29], [30]. Indeed, random fed and fasted blood glucose concentrations are significantly decreased in the Mbd5-knockout mice compared with the controls (Figure 5A, S4A). Concomitantly, the knockout mice perform better in the OGTT (Figure 5C, S4B), exhibit decreased insulin concentrations (Figure 5B) and showed improved performance in the ITT (Figure 5D). Improved hepatic insulin sensitivity in the absence of Mbd5 is also confirmed by an increase in Akt activation (phosphorylation) with or without insulin treatment (Figure 5E). Moreover, the enhanced glycolysis found in the liver further confirms the elevated insulin sensitivity that was observed in the mutant mice (Figure 5F, G). The reduction in perigonadal and subcutaneous fat (Figure 2E) also suggests improved insulin action in the mutant mice. Mouse model of conditional knockout of Mbd5 in liver or adipose tissues will be needed to elucidate the relationship between Mbd5 and obesity associated insulin resistance. Overall, the elevated insulin sensitivity and the resulting persistant hypoglycemia might contribute to postnatal lethality of the knockout mice.

The mechanism underlying the growth defect and the altered glucose homeostasis in Mbd5-knockout mice remains an area of future study. All known MBD family proteins can mediate silencing of gene expression by recruiting chromatin remodeling co-repressor complexes [8]. Although MBD5 cannot bind methylated DNA directly, it associates with heterochromatin [12]. MBD5 might bind to DNA in a complex as it interacts directly with myocyte enhancer-binding factor 2C (MEF2C) [34], which plays a crucial role in development, neurogenesis and neuronal gene regulation [35]. The haploinsufficiency of *MEF2C*, associated with a microdeletion in 5q14.3, results in severe mental retardation [36], which is strikingly similar to the phenotype of *MBD5* deficiency. Thus, MBD5 and MEF2C might function in the same signaling pathway to regulate the expression of key neurodevelopmental genes. MBD5 might also associate with a histone deacetylase to execute its function because the MBD family protein can mediate repression through their association with HDAC complexes. Further studies using conditional knockout mice will be needed to identify the mechanism of Mbd5-dependent regulation of somatic growth and glucose homeostasis.

In summary, our study uncovered a novel role for the MBD family protein MBD5 in the control of somatic growth and glucose homeostasis. The Mbd5*-*mutant mice partially mimic the developmental delay and growth retardation of human patients with 2q23.1 microdeletion syndrome. Therefore, the mice generated in this study will be useful for further studies aimed at elucidating of the pathogenic and molecular mechanism underlying this syndrome. Furthermore, Mbd5 deficiency results in a phenotype that has previously been connected to healthy aging (increased insulin sensitivity accompanied by reduced glucose, insulin, and GH levels). Thus, further analysis of Mbd5-knockout mice with respect to longevity might provide new insights into the connections between metabolism, growth and aging.

## Materials and Methods

### Ethics Statement

In this study, the experimental procedures for the use and care of animals were approved by the Ethics Committee of Shanghai Institutes for Biological Sciences, Chinese Academy of Sciences.

### Construction of the *Mbd5* Targeting Vector and the Generation of Knockout Mice

Standard recombineering method was used to generate an *Mbd5* targeting vector [37]. Briefly, the DNA fragment covering the *Mbd5* gene was retrieved into the pL253 vector from BAC clone bMQ102M9 (Wellcome Trust Sanger Institute, Hinxton, Cambridge, UK). An N-terminal coding region of *Mbd5*, which included a 1.5-kb fragment covering the first exon, was floxed by *lox*P sites that were introduced from the pL452 and pL451 vectors. The targeting vector was linearized with *Not*I and electroporated into MPI-II ES cells (129Sv/Pas derived); these transformed cells were subsequently cultured in the presence of G418 and ganciclovir on mitotically inactivated MEF cells. The targeted ES clones were identified by Southern blotting using the 5′ and 3′ external probes which were prepared by PCR (5′ probe forward, 5′-AACTGATAGGTCACAAGTTCTTCA-3′; reverse, 5′-AATTCCAGGGGCTTGATTTT-3′; 3′ probe forward, 5′-CCGTAGATGGCATGGAAATAAT-3′; reverse, 5′-GGTGCTCAGATACACAGACAGC-3′). Positive ES clones were microinjected into the C57BL/6J blastocysts and then transferred into pseudopregnant foster mothers. Chimeric male mice were bred with C57BL/6J females to generate *Mbd5^f/+^* mice. To generate homozygous Mbd5*-*knockout mice, *Mbd5^f/+^* mice were crossed with *EIIa-Cre* mice in which Cre was expressed in the embryo, and the resulting offspring were intercrossed to generate *Mbd5^−/−^* mice. To generate brain-specific *Mbd5* null mice, *Mbd5^+/−^* mice were crossed with *Nestin-Cre* mice [27], and the [*Mbd5^+/−^*, Nestin-Cre] pups were crossed with *Mbd5^f/f^* mice. The [*Mbd5^f/−^*, Nestin-Cre] mice (BSKO) had *Mbd5^−/−^* genotype in the brain. Mouse genotypes were identified by PCR analysis of tail DNA samples using the following primers: P1, 5′- CAGGTCATAAAAGTTGGTGGGTA-3′; P2, 5′- CTGCTCTAAATCCCTGGCCTTC-3′; P3, 5′- TTAAGCTTCCACTTCATTTTCCA3′; Nestincre-F, 5′-TCATGAGGATTCCAACACCA-3′; Nestincre-R, 5′- AATGTTGCTGGATAGTTTTTACTGC-3′. Normal littermate controls were used with the same age in all experiments.

### Tissue Collection, RNA Extraction and Quantitative Real-time PCR

The mice were euthanized, and harvested tissues were snap-frozen in liquid nitrogen. The total RNA was purified using TRIzol reagent following the manufacturer’s protocol (Invitrogen), and the cDNA was synthesized from 1 µg of total RNA using Primescript RT Reagent kit with genomic DNA Eraser (TAKARA). The mRNA levels were measured by quantitative real-time PCR using SYBR Green reagents (Biotium). All primer information is shown in Table S2. The relative level of transcripts of interest was calculated by first normalizing to a housekeeping gene (*β-actin* or *Gapdh*) and then normalizing to wild-type controls using the 2^−ΔΔCT^ method.

### Glucose Measurement, OGTT and ITT

Blood glucose levels in blood samples from tail vein were measured using the OptiumXceed glucometer (Abbott). The OGTT (1 g/kg of body weight, oral) was performed in the fasting state, and the ITT (0.8 IU/kg of body weight, intraperitoneal) was performed in the fed state. The blood glucose was measured at the indicated time points using a glucometer.

### Hormone Measurement

Orbital blood was collected and serum was prepared for hormone measurements. ELISA kits for measuring GH (Millipore), IGF-1 (R&D Systems) and insulin (Mercodia) concentrations were used according to the manufacturer’s instruction. For pituitary GH determination, pituitaries were sonicated in PBS supplemented with protease inhibitors (Roche).

### Histological Analysis

Paraffin-embedded pancreatic tissues (sectioned at a 5-µm thickness) and pituitary cryosection (at an 8-µm thickness) were processed for hematoxylin and eosin (H & E) staining [38].

### Antibodies and Immunofluorescence Microscopy

The monoclonal anti-insulin and anti-glucagon antibodies were from Sigma-Aldrich. The polyclonal anti-GLUT2 antibody was from Abcam. The Alexa-Fluor 488 goat anti-rabbit IgG and Alexa-Fluor 546 goat anti-mouse IgG were purchased from Molecular Probes. The tissues for immunofluorescence were embedded in OCT and the cyrosections were blocked in 1% BSA and 0.3% Triton X-100 in PBS. Then, sections were incubated sequentially with primary and fluorochrome-labeled secondary antibodies. After DAPI staining, the sections were mounted with mount gel (Invitrogen™), and images of the stained sections were captured with a Leica TCS SP2 laser confocal microscope or Olympus BX51 microscope.

### Western Blotting

Liver tissues were homogenized in lysis buffer (20 mM Tris-HCl, pH 7.4, 137 mM NaCl, 1 mM CaCl_2_, 1 mM MgCl_2_, 1% NP-40, 0.1 mM sodium orthovanadate, 0.1 mM sodium fluoride and protease inhibitor cocktails (Roche)). The homogenates were centrifuged at 20,000 g at 4°C for 15 min, and the supernatant was collected. Western blot analysis was carried out with antibodies against pan-Akt, p-Akt (S473 and T308) (Cell Signaling Technology) and α-tubulin (Sigma).

### Lactate Assay

The lactate concentration was determined with a Lactate Assay Kit (BioVision). The optical density (OD) at 570 nm was measured 30 min after the addition of substrate.

### Statistical Analysis

The number of mice in each experimental group is indicated in the figure legends. A two-tailed Student’s t-test was used to calculate P-values. All values are presented as means ± standard error (SEM). Differences were considered significant if P<0.05.

## Supporting Information

Figure S1 Additional phenotypic characterization of Mbd5 mutant mice. (A) Body weight of male wild-type (+/+) and heterozygous (+/−) mice at 8 weeks of age. n = 6 per group. (**B**) Comparable milk was present in the dead knockout (KO) pups and the corresponding-sacrificed wild-type littermates (WT). (**C**) Brain from wild-type (WT) and knockout (KO) mice at P14.
(TIF)

Figure S2 Additional pituitary analysis of Mbd5 deficient mice. (**A**) Serum GH concentrations in 2-week-old female knockout mice and littermate controls. n = 4–5 per genotype. (**B**) Real-time PCR analysis of pituitary expression of *Ghrhr*, *Ghsr*, *Sstr2* and *Sstr5* in female knockout mice and their littermate controls at P14. n = 5 per genotype. (**C**) Morphology of the pituitary tissues from P14 WT and KO mice at 40× magnification. The cryosections were stained with hematoxylin and eosin. The KO pituitaries show no overt histological abnormalities. AP: anterior pituitary; PP: posterior pituitary. (**D**) Unchanged expression of *TSHβ* and *ACTH* in Mbd5-deficient pituitary as measured by real-time PCR analysis. *Gapdh* was used for normalization. At least 5 pairs of matched female mice were used for the comparison.
(TIF)

Figure S3 Reduced circulating IGF-1 level in newborn Mbd5-deficient mice. (**A**) Comparison of *Igf-1* mRNA levels in hypothalamus, pituitary, muscle, and epigonadal adipose tissues between knockout mice and their littermate controls at P14. n = 5 per group. (**B**) Serum IGF-1 concentrations were measured in wild-type (+/+), heterozygous (+/−), and homozygous (−/−) mice at the newborn stage. For each group, n = 7; *, P<0.05; **, P<0.01.
(TIF)

Figure S4 Additional analysis of glucose homeostasis of Mbd5 knockout mice. (**A**) Fasted blood glucose level in control and Mbd5-knockout male mice at P14. (**B**) The OGTT of control and Mbd5-knockout male mice at P14. Basal glucose concentration at time 0 was set as 100%. For each group, n = 5. *, P<0.05; **, P<0.01.
(TIF)

Figure S5 Normal pancreatic development in Mbd5-deficient mice. (**A**) Morphology of the pancreas at P14. The paraffin-embedded sections were stained with hematoxylin and eosin. Scale bar: 200 µm. (**B**) Normal distribution of insulin- and glucagon-expressing cells in the pancreas of Mbd5-deficient mice. Representative images of pancreatic cryosections of wild-type and Mbd5*-*knockout mice at P9 are shown. The sections were stained using antibodies against insulin and glucagon, respectively. Scale bar: 50 µm. (**C**) Distribution of the GLUT2 transporter in the pancreases of Mbd5-deficient mice at P14. The cryosections were stained using an anti-GLUT2 antibody. Insulin staining was used to distinguish the islets. Scale bar: 50 µm.
(TIF)

Table S1 Fertility of *Mbd5^+/−^* mice.
(DOCX)

Table S2 List of oligonucleotide primers used in quantitative real-time PCR.
(DOCX)

Movie S1 Behavior of wild-type mice. Shown in the video is the movement behavior of representative wild-type mice at age P14.
(MP4)

Movie S2 Behavior of Mbd5-deficient mice. Shown in the video is the movement behavior of representative Mbd5 knockout mice at age P14.
(MP4)
