# Supplementary figures and images for: The Essential Role of Mbd5 in the Regulation of Somatic Growth and Glucose Homeostasis in Mice
Source: PLoS One. 2012 Oct 15;7(10):e47358. doi: 10.1371/journal.pone.0047358 (PMC3471830; doi:10.1371/journal.pone.0047358)

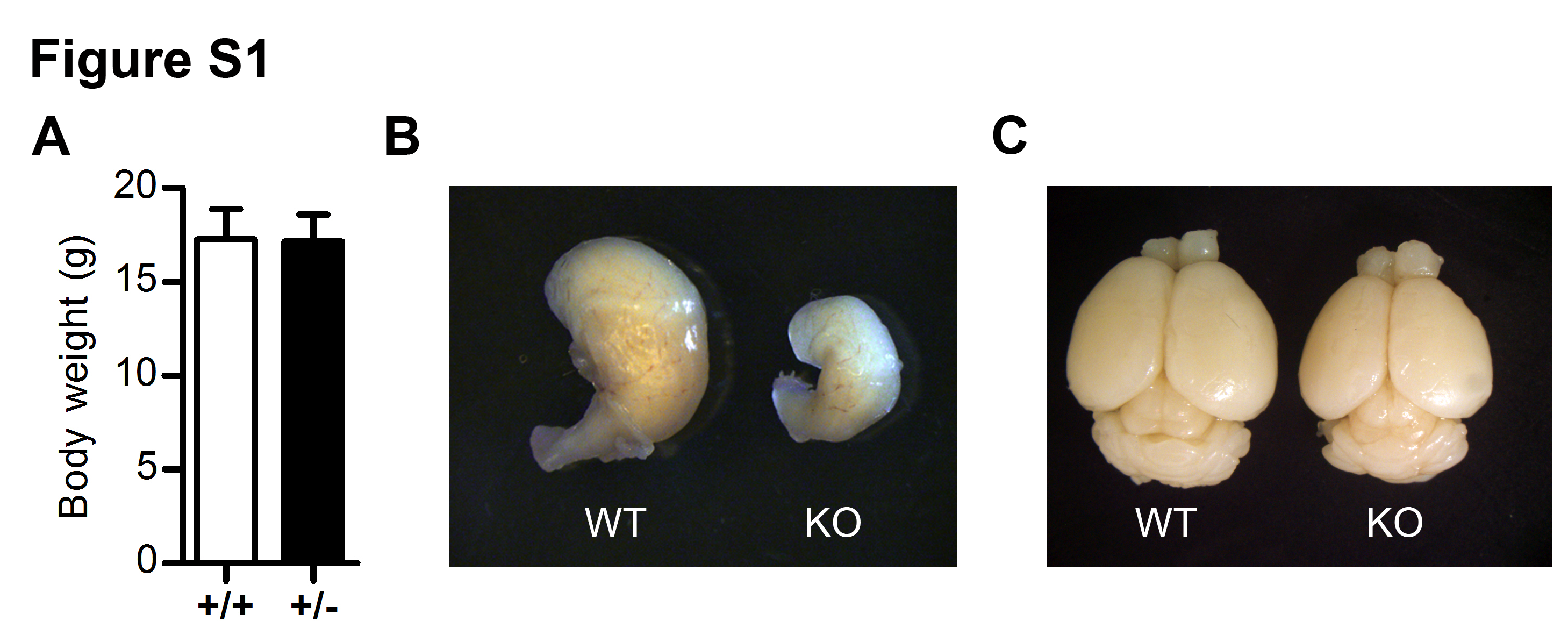

Supplement: Figure S1 — Additional phenotypic characterization of Mbd5 mutant mice. (A) Body weight of male wild-type (+/+) and heterozygous (+/−) mice at 8 weeks of age. n = 6 per group. (B) Comparable milk was present in the dead knockout (KO) pups and the corresponding-sacrificed wild-type littermates (WT). (C) Brain from wild-type (WT) and knockout (KO) mice at P14. (TIF) [file pone.0047358.s001.tif]

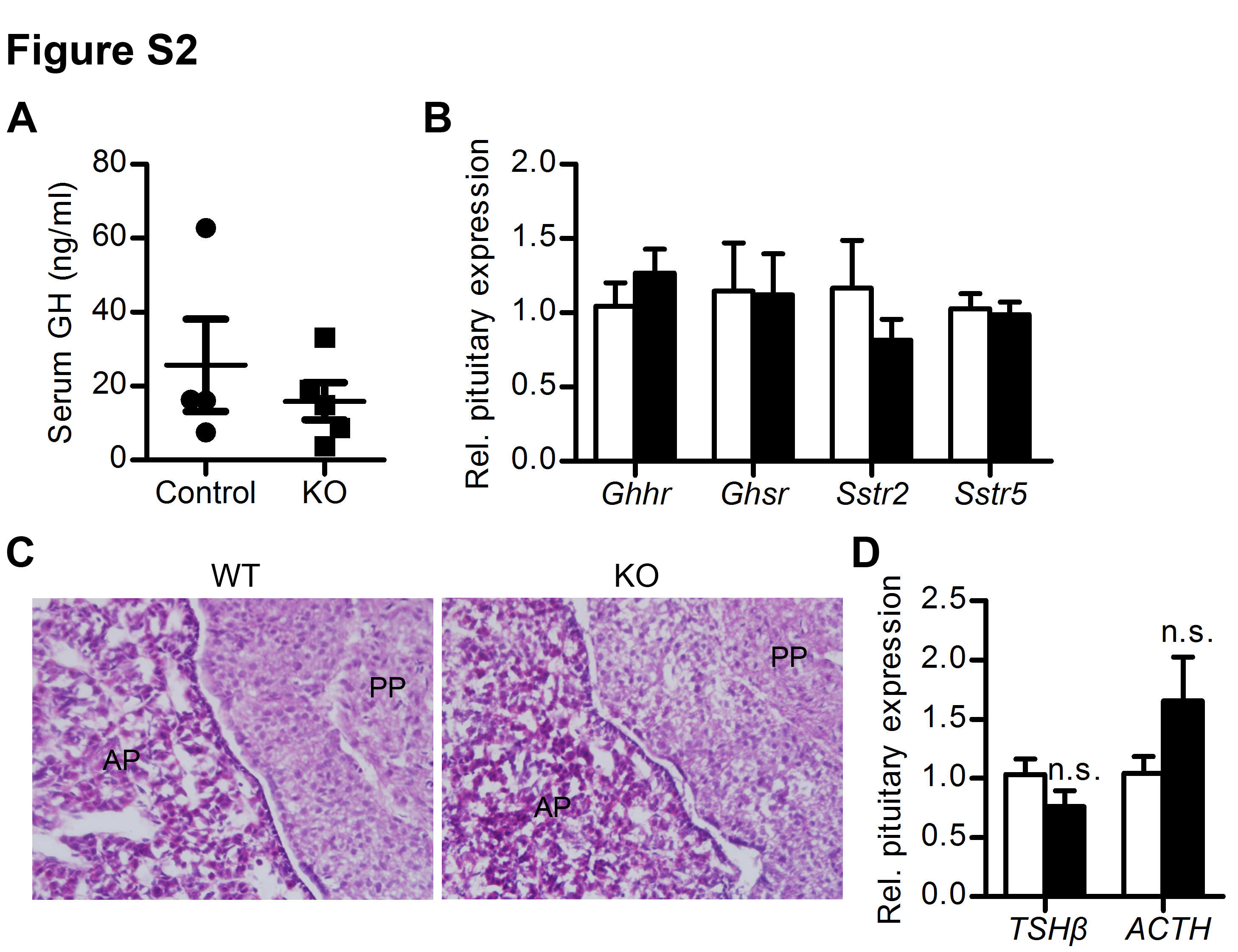

Supplement: Figure S2 — Additional pituitary analysis of Mbd5 deficient mice. (A) Serum GH concentrations in 2-week-old female knockout mice and littermate controls. n = 4–5 per genotype. (B) Real-time PCR analysis of pituitary expression of Ghrhr, Ghsr, Sstr2 and Sstr5 in female knockout mice and their littermate controls at P14. n = 5 per genotype. (C) Morphology of the pituitary tissues from P14 WT and KO mice at 40× magnification. The cryosections were stained with hematoxylin and eosin. The KO pituitaries show no overt histological abnormalities. AP: anterior pituitary; PP: posterior pituitary. (D) Unchanged expression of TSHβ and ACTH in Mbd5-deficient pituitary as measured by real-time PCR analysis. Gapdh was used for normalization. At least 5 pairs of matched female mice were used for the comparison. (TIF) [file pone.0047358.s002.tif]

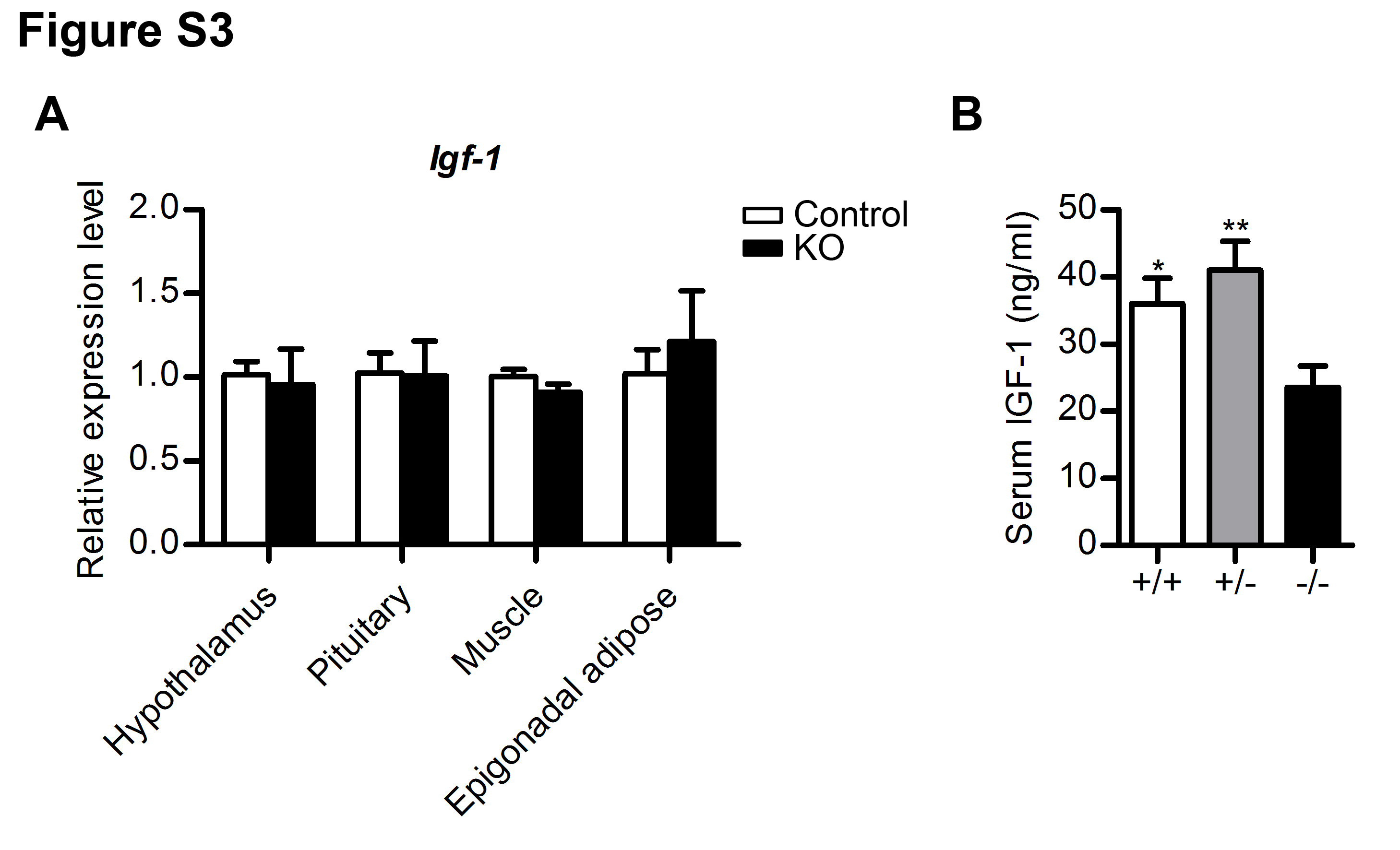

Supplement: Figure S3 — Reduced circulating IGF-1 level in newborn Mbd5-deficient mice. (A) Comparison of Igf-1 mRNA levels in hypothalamus, pituitary, muscle, and epigonadal adipose tissues between knockout mice and their littermate controls at P14. n = 5 per group. (B) Serum IGF-1 concentrations were measured in wild-type (+/+), heterozygous (+/−), and homozygous (−/−) mice at the newborn stage. For each group, n = 7; *, P<0.05; **, P<0.01. (TIF) [file pone.0047358.s003.tif]

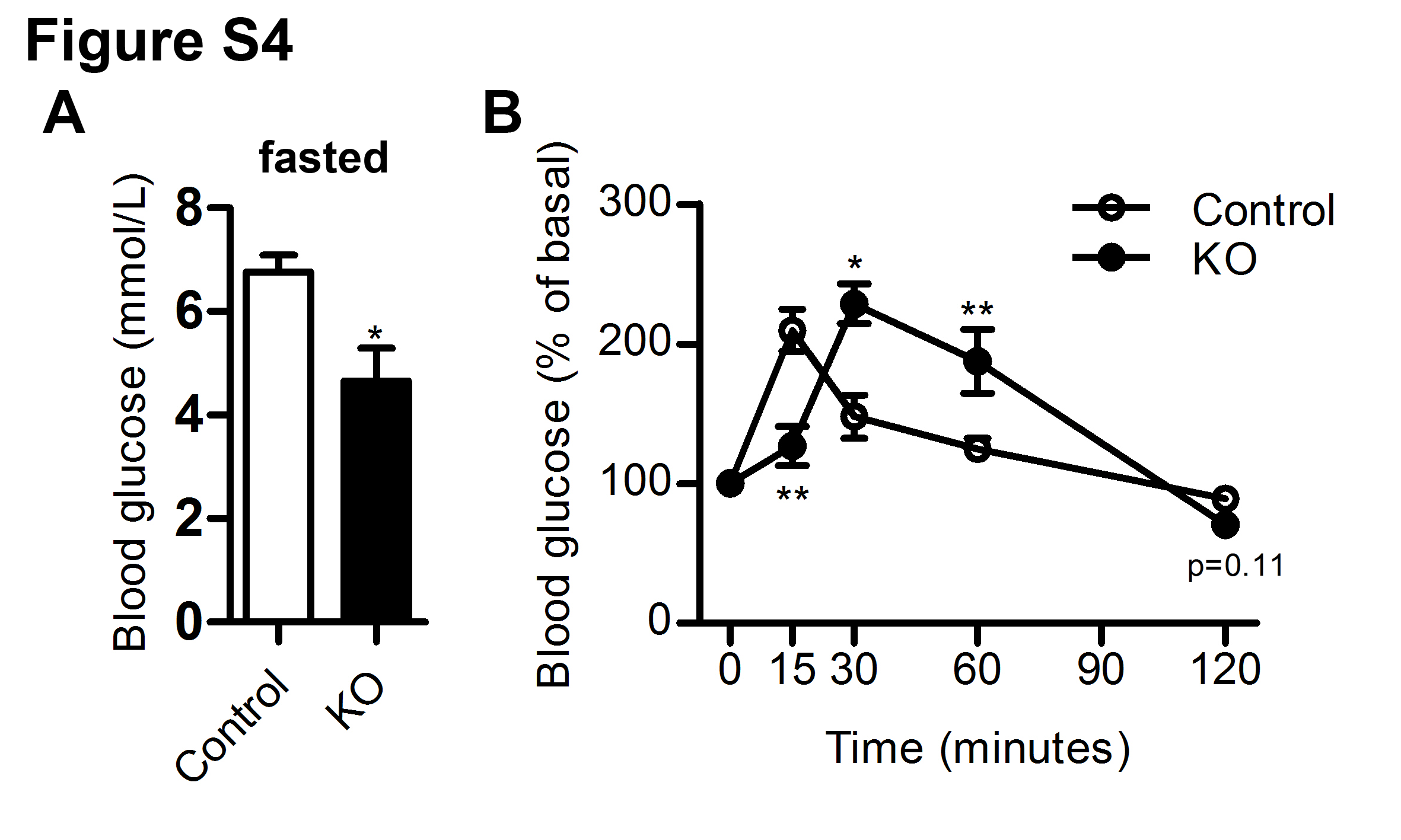

Supplement: Figure S4 — Additional analysis of glucose homeostasis of Mbd5 knockout mice. (A) Fasted blood glucose level in control and Mbd5-knockout male mice at P14. (B) The OGTT of control and Mbd5-knockout male mice at P14. Basal glucose concentration at time 0 was set as 100%. For each group, n = 5. *, P<0.05; **, P<0.01. (TIF) [file pone.0047358.s004.tif]

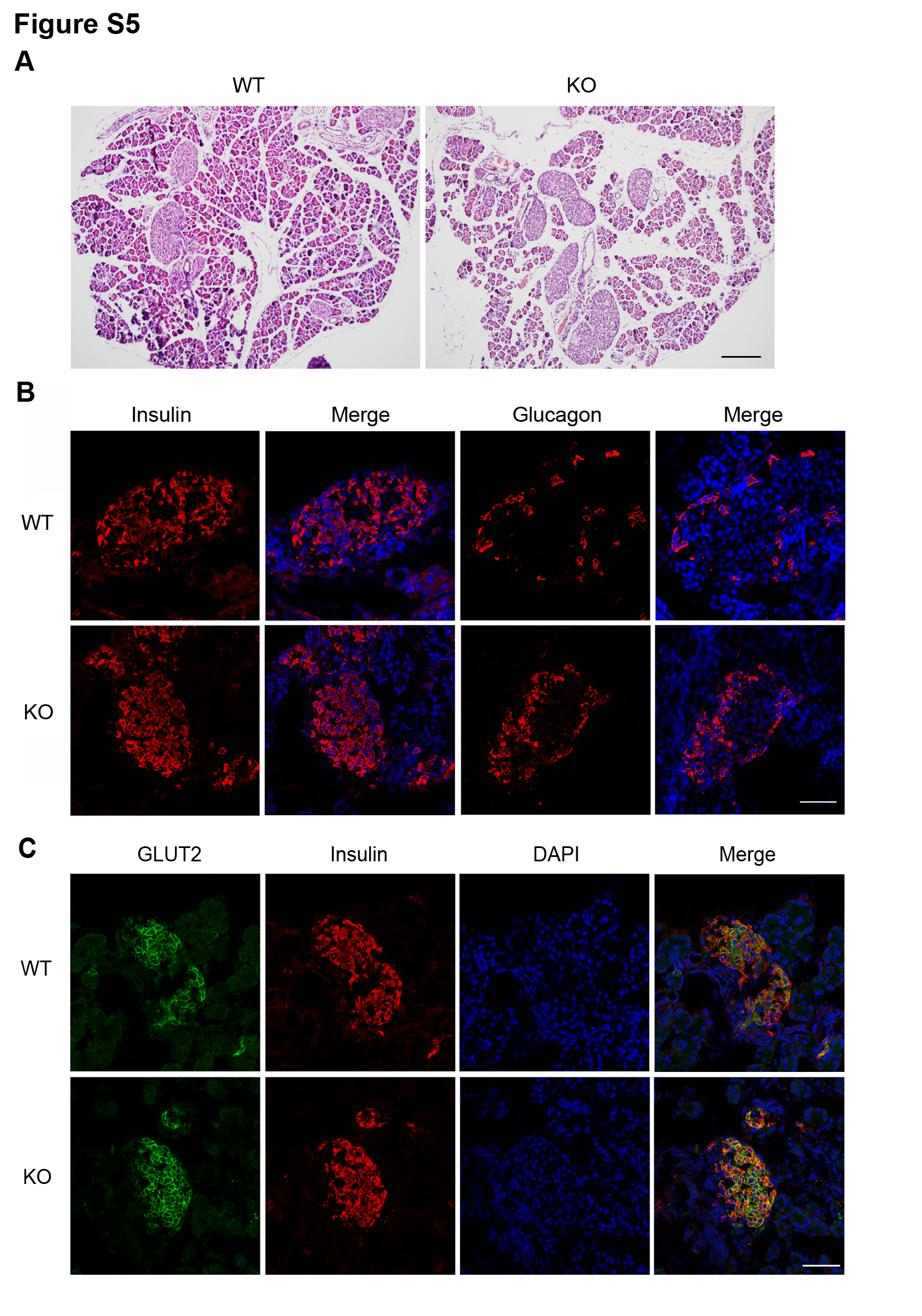

Supplement: Figure S5 — Normal pancreatic development in Mbd5-deficient mice. (A) Morphology of the pancreas at P14. The paraffin-embedded sections were stained with hematoxylin and eosin. Scale bar: 200 µm. (B) Normal distribution of insulin- and glucagon-expressing cells in the pancreas of Mbd5-deficient mice. Representative images of pancreatic cryosections of wild-type and Mbd5-knockout mice at P9 are shown. The sections were stained using antibodies against insulin and glucagon, respectively. Scale bar: 50 µm. (C) Distribution of the GLUT2 transporter in the pancreases of Mbd5-deficient mice at P14. The cryosections were stained using an anti-GLUT2 antibody. Insulin staining was used to distinguish the islets. Scale bar: 50 µm. (TIF) [file pone.0047358.s005.tif]
