# Supplementary material for: The Essential Role of Mbd5 in the Regulation of Somatic Growth and Glucose Homeostasis in Mice
Source: PLoS One. 2012 Oct 15;7(10):e47358. doi: 10.1371/journal.pone.0047358 (PMC3471830; doi:10.1371/journal.pone.0047358)
Supplement: Table S1 — Fertility of Mbd5+/− mice. (DOCX) [file pone.0047358.s006.docx]

**Table S1. Fertility of *Mbd5^+/-^* mice.**

| Mating pairs | No. of matings | No. of litters generated | Total No. of viable pups born | Average litter size (means±SEM) |
| --- | --- | --- | --- | --- |
| WT X WT | 7 | 7 | 63 | 9.0 ± 0.62 |
| *Mbd5^+/-^* X *Mbd5^+/-^* | 10 | 10 | 93 | 9.3 ±0.42 |

Number of mating was based on plugs seen.
