# Supplementary material for: The Essential Role of Mbd5 in the Regulation of Somatic Growth and Glucose Homeostasis in Mice
Source: PLoS One. 2012 Oct 15;7(10):e47358. doi: 10.1371/journal.pone.0047358 (PMC3471830; doi:10.1371/journal.pone.0047358)
Supplement: Table S2 — List of oligonucleotide primers used in quantitative real-time PCR. (DOCX) [file pone.0047358.s007.docx]

**Table S2. List of oligonucleotide primer pairs used in quantitative real-time PCR analysis.**

| Target Gene | Sense Primer (5’-3’) | Antisense Primer (5’-3’) |
| --- | --- | --- |
| *Gh* | GCTACAGACTCTCGGACCTC | CGGAGCACAGCATTAGAAAACAG |
| *Ghr* | ACAGTGCCTACTTTTGTGAGTC | GTAGTGGTAAGGCTTTCTGTGG |
| *Als* | GCGCTCTTGTGTGGCTTGG | GCTGTGATGAGGTTGCGGTC |
| *Igf-1* | CTGGACCAGAGACCCTTTGC | GGACGGGGACTTCTGAGTCTT |
| *Ghrhr* | GCGGAGTTACACTGGGTCAC | ATGCAAGCTCATCGTCTCTCA |
| *Ghsr* | TGGAGATCGCGCAGATCAG | CCGGGAACTCTCATCCTTCAG |
| *Sstr2* | CTGGAATCCGAGTGGGATCAT | ACGGAAGAGACGTTGAAGATGTA |
| *Sstr5* | GGCTTCCACACCTAGCTGG | AGCACAGGCACTAATACCGC |
| *Mbd5* | AGACAAGGAAGGAGGTCTTGC | GTGTGGCCACTGCAATGATCTTTCT |
| *TSHβ* | GGGCAAGCAGCATCCTTTTG | GTGTCATACAATACCCAGCACAG |
| *ATCH* | ATGCCGAGATTCTGCTACAGT | TCCAGCGAGAGGTCGAGTTT |
| *Pfk1* | TGTGGTCCGAGTTGGTATCTT | GCACTTCCAATCACTGTGCC |
| *Ldhb* | CATTGCGTCCGTTGCAGATG | GGAGGAACAAGCTCCCGTG |
| *Pdk4* | AGGGAGGTCGAGCTGTTCTC | GGAGTGTTCACTAAGCGGTCA |
| *β-actin* | GAAATCGTGCGTGACATCAAAG | TGTAGTTTCATGGATGCCACAG |
| *Gapdh* | GCCAGCCTCGTCCCGTAGACA | CAACAATCTCCACTTTGCCACTGC |
